# Season-specific nitrogen management shapes soil microbial communities and crop productivity in wheat–maize rotation system

**DOI:** 10.3389/fpls.2026.1938392

**Published:** 2026-09-16

**Authors:** Mengru Xue, Wenyang Li, Haosen Wei, Zerong Wang, Baojin Guo, Chuangyun Wang, Peng Zhou, Fei Gao

**Affiliations:** 1 College of Agriculture, Shanxi Agricultural University, Taiyuan, Shanxi, China; 2 Institute of Crop Science, Chinese Academy of Agricultural Sciences, Beijing, China

**Keywords:** cross-seasonal effects, nitrogen fertilizer use efficiency, seasonal N carryover, soil bacteria–fungi communities, soil-crop system coordination, winter wheat–summer maize rotation

## Abstract

Based on a long-term positioned field experiment in a wheat-maize double-cropping system, the regulatory mechanisms of nitrogen fertilizer legacy on soil-crop coordination and microbial functions remain unclear. A two-year field experiment was conducted with four treatments: CK: no N fertilizer applied during either wheat or maize seasons; W1M0: N carryover only during the winter wheat season; W0M1: N carryover only during the summer maize season; W1M1: N carryover during both wheat and maize seasons. Compared with the unfertilized control (CK), W1M0 and W0M1 increased average annual yields by 45.7% and 42.2%, respectively, while W1M1 increased by 58.6%. On average, W1M1 produced the highest yield (21.8 t ha^-1^), with W1M0 and W0M1 achieving 91.9% and 89.7% of W1M1 yield, respectively. fertilization increased the abundance of predicted chemoheterotrophic functions. W0M1 influenced predicted N-transformation functions and fungal guilds. Bacterial community composition responded more strongly than fungal communities. Soil-crop coupling coordination varied dynamically with growth stages and N management patterns. PLS-SEM revealed that soil nitrogen content from the preceding wheat season explained 97.5% of the variance in subsequent maize soil physicochemical properties, which was 2.65 times higher than the corresponding explanatory power of maize-season N (36.8%), indicating a stronger predictive legacy of wheat-season N application on soil-crop coordination. In conclusion, seasonal N coordination is more crucial than total N input. Leveraging the superior legacy effect of wheat-season N application provides a scientific basis for precise fertilization regulation in rotation systems.

## 1 Introduction

The winter wheat-summer maize rotation system constitutes a predominant double-cropping pattern on the North China Plain, serving as a crucial component in the semi-arid to semi-humid regions of northern China (Sha et al., 2023). To sustain high crop productivity, intensive fertilizer application has become a common management practice in this system. However, excessive nitrogen (N) fertilization often decreases nitrogen use efficiency (NUE) and increases the accumulation of residual nutrients in agricultural soils, thereby generating potential environmental risks and reducing fertilizer utilization efficiency (Gu et al., 2023; Wang et al., 2023). Previous studies have shown that reducing N application rates to 180 kg N ha^-1^ can maintain stable crop yields in wheat-maize rotation systems under certain conditions (Duan et al., 2019), suggesting that residual nutrients from previous fertilization may contribute to subsequent crop production.

Residual fertilizer nutrients, particularly residual soil N, can persist across cropping seasons and influence nutrient availability and crop N uptake in subsequent crops (Kebede, 2021). In the North China Plain, residual N recovery rates have been reported to range from 36.52% to 50.42% for maize and from 29.2% to 33.6% for wheat (Zhang et al., 2018; Dang et al., 2003). Moreover, the recovery efficiency of residual N by subsequent crops exhibits substantial spatial and temporal variation. For example, Jia reported that the recovery efficiency of residual N by summer maize ranged from 5% to 10% in alluvial plain regions (Jia et al., 2011), whereas higher recovery rates (8%-17%) were observed on the Loess Plateau (Poffenbarger et al., 2018). Residual N uptake has also been detected in later rotation cycles, with measurable recovery efficiencies remaining in the third-season winter wheat and fourth-season summer maize crops (Dong et al., 2012). These findings indicate that fertilizer legacy effects may contribute to crop productivity beyond the season of fertilizer application.

Soil microbial communities are important regulators of nutrient cycling and soil biochemical processes in agricultural ecosystems (Li et al., 2023a). Previous studies have demonstrated that microbial community composition differs substantially between wheat and maize growing seasons, with fungal communities often exhibiting stronger seasonal variation than bacterial communities (Hartmann et al., 2014; Lu et al., 2022). Fertilization practices can alter soil physicochemical properties, including nutrient availability and pH, thereby reshaping microbial community structure and predicted ecological functions (Gaudel et al., 2024; Jiang et al., 2025). Nitrogen addition has also been associated with changes in N-cycling microbial groups, such as diazotrophs and ammonia-oxidizing microorganisms (Li et al., 2023b; Zhang et al., 2022). In rotation systems, seasonal crop succession further influences rhizosphere microbial assembly and microbial functional adaptation (Gfeller et al., 2023; Ma et al., 2022). Although previous studies have examined microbial responses to fertilization or crop rotation independently, the inter-seasonal legacy effects of season-specific fertilization on microbial community structure and predicted functions across wheat and maize seasons remain poorly understood.

In addition to microbial responses, evaluating the coordination between soil nutrient status and crop productivity is important for understanding the sustainability of fertilization strategies. The Soil-Crop System Coupling Coordination Degree (D) has been proposed as an indicator for assessing the synchronization between soil and crop subsystems (Han et al., 2025; Liu et al., 2026). In the present study, the soil subsystem was represented primarily by soil N status, whereas the crop subsystem was represented by crop yield performance. Therefore, the coupling coordination analysis was used to evaluate the degree of synchronization between soil N availability and crop productivity under different seasonal fertilization treatments.

Therefore, this study aimed to evaluate the effects of fertilizer legacy from different cropping seasons on crop yield, nitrogen use efficiency (NUE), soil microbial community structure, and predicted microbial functions in a wheat-maize rotation system. Specifically, the objectives were to: (1) determine how season-specific fertilization treatments influence microbial community structure and predicted functional succession during wheat and maize seasons; (2) identify differential microbial responses to fertilization legacy effects across cropping seasons; and (3) evaluate the associations among soil chemical properties, microbial community characteristics, predicted microbial functions, and crop productivity.

## 2 Materials and methods

### 2.1 Experimental design

This study was conducted from 2021 to 2023 at an experimental station (35.68°N, 111.18°E; 450 m altitude) in Baicun Village, Xinjiang County, Shanxi Province, China. The site is situated on the Loess Plateau and experiences a warm-temperate continental monsoon climate (**Supplementary Figure 1**). The mean annual precipitation and temperature are 539.5 mm and 13.1 °C, respectively. Monthly precipitation and temperature data from June 2021 to June 2023 are presented in **Figure 1**. The soil is classified as Cambisol, and the experimental plots had a uniform fertilization history. Key physicochemical properties of the topsoil (0–20 cm depth) at the initiation of the experiment are provided in **Supplementary Table 1**. Urea is used as the nitrogen fertilizer, potassium dihydrogen phosphate as the potassium fertilizer, and calcium superphosphate as the phosphorus fertilizer. For specific fertilization methods, see **Table 1**.

**Figure 1:**
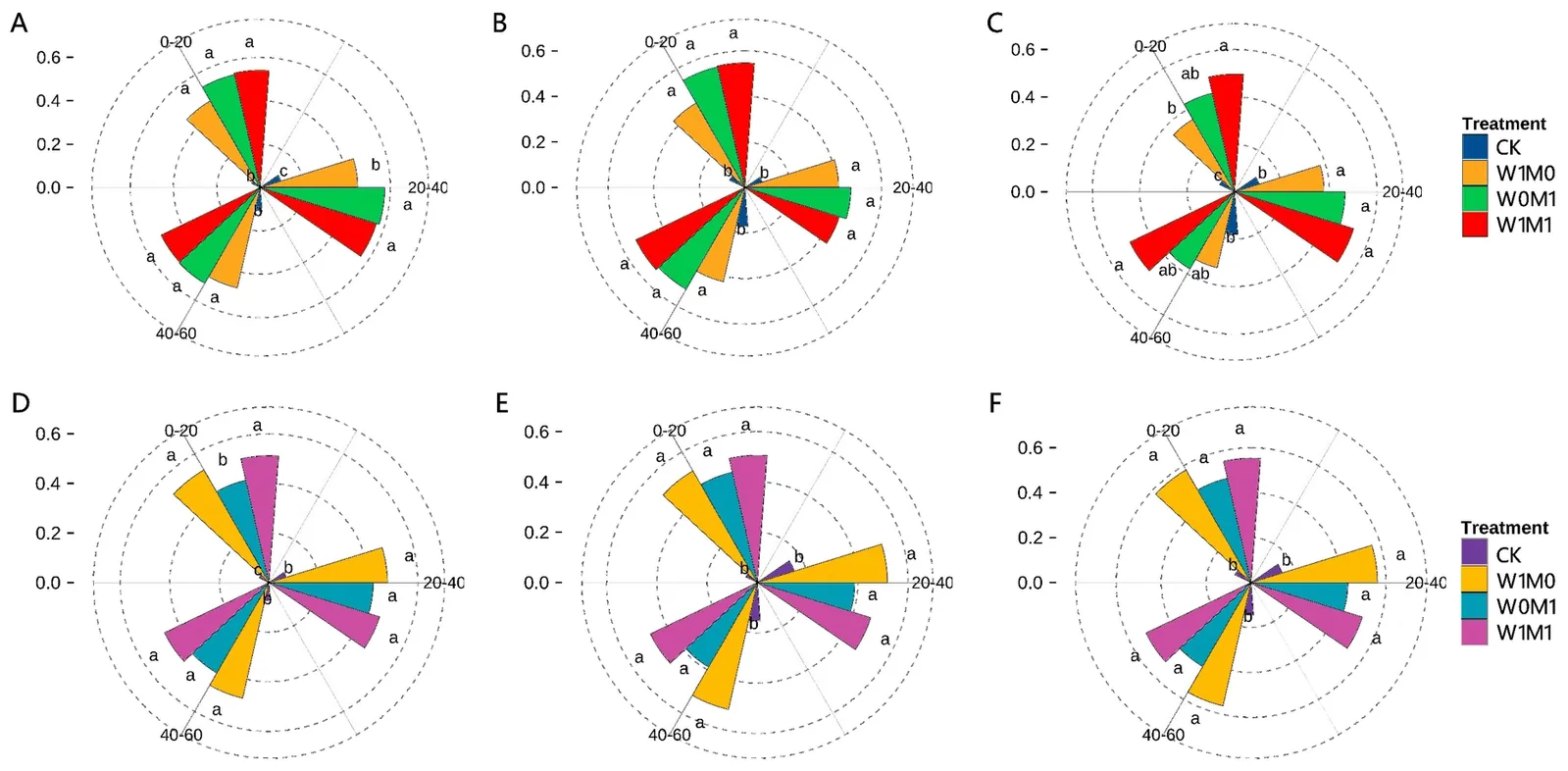
Effects of different seasonal N carryover application on the coupling coordination degree of soil-crop system in winter wheat and summer maize season. **(A–F)** represent the coupling coordination degree at different growth stages of winter wheat and summer maize. Specifically, **(A)** maize jointing stage; **(B)** maize tasseling stage; **(C)** maize physiological maturity stage; **(D)** wheat jointing stage; **(E)** wheat flowering stage; **(F)** wheat maturity stage. Each radial axis in the image represents the coupling coordination degree (D value) calculated for different soil layers (0–20 cm, 20–40 cm, and 40–60 cm). The coupling coordination degree (D) reflects the coordinated development level between the soil subsystem and crop subsystem. The value of D ranges from 0 to 1, with higher values indicating stronger coordination between soil N and crop systems. Different lowercase letters indicate significant differences among treatments within the same soil layer and growth stage (P < 0.05).

**Table 1:** Fertilization method in experimental field.

Treatment	Winter wheat season			Summer maize season		
	N (kg ha^-1^)	P_2_O_5_ (kg ha^-1^)	K_2_O (kg ha^-1^)	N (kg ha^-1^)	P_2_O_5_ (kg ha^-1^)	K_2_O (kg ha^-1^)
CK	0	150	37.5	0	150	37.5
W1M0	150	150	37.5	0	150	37.5
W0M1	0	150	37.5	150	150	37.5
W1M1	150	150	37.5	150	150	37.5

Four treatments were established: CK: no N fertilizer applied during either wheat or maize seasons; W1M0: N carryover only during the winter wheat season; W0M1: N carryover only during the summer maize season; W1M1: N carryover during both wheat and maize seasons. Phosphate and potash fertilizers were applied in all treatments. The experiment was arranged in a randomized complete block design with three replications. Each plot measured 8.0 × 8.0 m and was separated by a 1.0 m buffer zone. The same experimental plots were maintained throughout the entire study period.

The winter wheat cultivar used was ‘Liangxing 68’, and the summer maize cultivar was ‘Denghai 605’. Winter wheat (Triticum aestivum L.) was sown in mid-October and harvested in early June of the following year. Summer maize (Zea mays L.) was sown in mid-June and harvested in early October of the same year. Winter wheat was sown by drilling. The planting density for summer maize was maintained at approximately 67,500 plants per hectare.

### 2.2 Experiment material

All chemical reagents, including sodium hydroxide, concentrated sulfuric acid, potassium chloride, and hydrochloric acid, were of analytical grade and purchased from Tianjin Kemiou Chemical Reagent Co., Ltd. Hydrogen peroxide was obtained from Tianjin Ouboke Chemical Co., Ltd.

Instrumentation: The following instruments were used in this study: an AA3 continuous flow analyzer (SEAL Analytical, Germany); K9860 Fully automatic Kjeldahl nitrogen analyzer (Hanon, China) and a digestion system (Siping, China).

### 2.3 Sampling and measurement

#### 2.3.1 Crop yield

Crop yield was determined at physiological maturity using a representative sampling method. For winter wheat, the number of productive panicles was counted from three randomly placed 1 m² quadrats per plot. Winter wheat grain yield was then calculated on an area basis and adjusted to a standard moisture content of 14%; summer maize grain yield was calculated on an area basis and adjusted to a standard moisture content of 12% (Liu et al., 2025). For summer maize, the number of productive ears was counted from three 1 m row sections per plot and converted to ears per square meter based on a row spacing of 0.6 m. Afterwards, the yields were calculated as follows:


$$Wheat/Maize\;ears\;per\;ha\;=\;Mean\;ears\;per\;1\;m^{2}\times 10000$$

$$Yield\;(kg\;ha^{-1})\;=\;\frac{\left(Ears\;per\;1\;ha\;\times \;Grains\;/ear\;\times \;1000-grain\;weight\right)}{100,000}$$

$$Annual\;yield\;(t\;ha^{-1})=\frac{\left(Summer\;Maize\;Yield+Winter\;Wheat\;Yield\right)}{1000}$$

#### 2.3.2 Soil sample collection

##### 2.3.2.1 Microbial determination of soil samples

In 2022, soil samples were collected during two distinct agricultural seasons, namely, winter wheat harvest (June 15) and summer maize maturity (October 10), using a five-point sampling method. Each treatment was replicated thrice. The soil samples were collected from depths of 0–20 cm. After removing stones and root debris, the samples were stored on ice, sieved (2 mm mesh) and preserved at -80 °C for DNA extraction.

##### 2.3.2.2 Soil nitrogen determination of soil samples

During each growth stage (winter wheat season: WJ (wheat joint stage), WF (wheat flower stage), and WH(wheat harvest stage); summer maize season: V6 (maize jointing stage), V12 (twelfth leaf collar stage), VT (maize tasseling stage), R3 (maize milk stage), and R6 (maize physiological maturity stage)), soil samples were collected from the 0-20, 20-40, and 40–60 cm soil layers. Samples were air-dried and sieved prior to analysis. Soil total nitrogen (TN) was determined using the H_2_SO_4_-H_2_O_2_ digestion method (Wang et al., 2024). Briefly, air-dried soil samples were finely ground and passed through a 0.149 mm sieve. Approximately 0.2-0.5 g of soil was digested with concentrated sulfuric acid (H_2_SO_4_) and hydrogen peroxide (H_2_O_2_) under controlled heating conditions until the solution became clear. The digest was then analyzed for total nitrogen content using the Kjeldahl distillation and titration method (or an automatic Kjeldahl nitrogen analyzer, if applicable). All measurements were expressed on an oven-dry soil basis (g kg^-1^). The following indices were calculated to assess nitrogen use efficiency (All nitrogen application rates are annual rates):


Annual N recovery efficiency (AREN, %) = (N−applied area plant nitrogen accumulation – No−N area plant nitrogen accumulation)/Nitrogen application rate × 100

$$Annual\;N\;partial\;factor\;productivity\;\left(APFPN,\;kg\;kg^{-1}\right)\;=\;Grain\;yield/Nitrogen\;application\;rate$$

$$N\;agronomic\;efficiency\;\left(AEN,\;kg\;kg^{-1}\right)\;=\;\left(Grain\;yield\;in\;fertilized\;area\;-\;Grain\;yield\;in\;unfertilized\;area\right)/Nitrogen\;application\;rate$$

$$\begin{array}{l} Annual\;N\;physiological\;efficiency\;\left(APEN,\;kg\;kg^{-1}\right)\;=\;\\ \left(Grain\;yield\;in\;nitrogen-applied\;area\;-\;Grain\;yield\;in\;nitrogen-unapplied\;area\right)/\\ \left(Plant\;nitrogen\;accumulation\;in\;nitrogen-applied\;area\;-\;Plant\;nitrogen\;accumulation\;in\;nitrogen-unapplied\;area\right) \end{array}$$

Nitrogen Harvest Index (NHI) = N accumulation at maize(wheat) grain/Nitrogen accumulation in above−ground biomass at maturity

The annual nitrogen input was 150 kg N ha^-1^ for single-season N treatments (W1M0 and W0M1) and 300 kg N ha^-1^ for the continuous N treatment (W1M1), allowing comparison between seasonal distribution patterns under reduced total N input and conventional continuous application. Phosphorus and potassium inputs were kept consistent across all treatments.

#### 2.3.3 Soil genomic DNA extraction and Illumina MiSeq sequencing

DNA was extracted from the samples using the CTAB-SDS (Cetyl Tri-methyl Ammonium Bromide-SDS) method (Turroni et al., 2011). The quality and concentration of the extracted DNA were verified by 1% agarose gel electrophoresis. The DNA was then diluted with sterile water to a concentration of 1 ng/μL^-1^ and used as the template for PCR amplification.

The V4 region of the bacterial 16S rRNA gene was amplified with the primers 515F (5’-CCTAYGGGRBGCASCAG-3’) and 806R (5’-GGACTACNNGGGTATCTAAT-3’). For fungal sequencing, the ITS1 region of the ITS rRNA gene was targeted using primers ITS5-1737F (5’-GAAGTAAAAGTCGTAACAAGG-3’) and ITS2-2043R (5’-GCTGCGTTCTTCATCGATGC-3’).

The PCR protocol consisted of initial denaturation at 98 °C for 1 min, followed by 30 cycles of denaturation at 98 °C for 15 s, annealing at 50 °C for 30 s, and extension at 72 °C for 30 s, with a final extension at 72 °C for 5 min. The PCR products were visualized on a 2% agarose gel to confirm amplification. Target fragments were excised and purified using the QIAquick Gel Extraction Kit (Qiagen, Germany). Purified amplicons were sequenced on an Illumina NovaSeq 6000 platform (Beijing Novogene Technology Co., Ltd).

### 2.4 Soil nitrogen-crop system coupling coordination degree analysis

#### 2.4.1 Establishment of evaluation indicator system

Soil nitrogen Subsystem (S): Soil total nitrogen (TN) in each soil layers (0–20 cm, 20–40 cm, 40–60 cm).

Crop Subsystem (C): Crop yield.

#### 2.4.2 Calculate the subsystem composite index

Employ the entropy weighting method to derive the subsystem’s composite performance index:


$$U_{S}=\sum_{i=1}^{n}\omega_{i}^{s}x_{i}^{s},\;\;U_{C}=\sum_{i=1}^{n}\omega_{j}^{s}x_{j}^{s}$$

where ω represents the indicator weight, U_S_ represents the composite index of each soil layer, and U_C_ represents the composite index of yield, χ and denotes the normalized indicator value. U_S_ and U_C_ are normalized (Liu et al, 2021). In this formula, Us are TN (0–20 cm), TN (20–40 cm) and TN (40–60 cm).

#### 2.4.3 Calculating coupling degree (C)

Coupling degree measures the strength of interaction between two subsystems. The commonly used formula is:


$$C=\frac{U_{S}\times U_{C}}{{\left(\alpha U_{S}+\beta U_{C}\right)}^{2}}$$

where α and β are the weights of the subsystems, set to 0.5. The value range of C is 0 to 1.0. The closer C is to 1, the tighter the coupling.

#### 2.4.4 Calculate the coordination index (T)

The Coordination Index reflects the overall development level of the subsystem:


$$T=\alpha U_{S}+\beta U_{C}$$

#### 2.4.5 Calculate the coupling coordination index (D)

The Coupling Coordination Index comprehensively evaluates both the coupling degree and the overall development level, serving as the final assessment metric:


$$D=\sqrt{C\times T}$$

The value of D is also within the range of 0 to 1.0; A higher value indicates a greater degree of association, coordination, and comprehensive development among subsystems.

### 2.5 Bioinformatic analysis and functional prediction

Raw paired-end reads were demultiplexed according to barcode sequences and merged using FLASH v1.2.7. Primer sequences were removed, and low-quality reads were filtered using fastp to obtain high-quality sequences. Chimeric sequences were identified and removed using VSEARCH, and the resulting effective tags were used for downstream analyses.

For bacterial communities, 16S rRNA gene sequences were clustered into operational taxonomic units (OTUs) at 97% sequence similarity using the UPARSE pipeline. Representative sequences of bacterial OTUs were taxonomically classified against the SILVA database (Release 123). Fungal ITS sequences were processed using the same OTU clustering strategy and annotated against the UNITE database (Version 8.2).

To reduce sequencing-depth bias, all samples were rarefied to the minimum sequencing depth before diversity analyses. Alpha diversity indices, including ACE, Chao1, Shannon, and Simpson indices, were calculated using QIIME and Mothur. Beta diversity was evaluated based on Bray-Curtis dissimilarity matrices and visualized using principal coordinate analysis (PCoA). Differences in microbial community composition among treatments were assessed using permutational multivariate analysis of variance (PERMANOVA) with 999 permutations.

Redundancy analysis (RDA) was conducted to evaluate the relationships between microbial community structures and environmental variables, and statistical significance was assessed using Monte Carlo permutation tests with 999 permutations.

Bacterial functional profiles were predicted using the Functional Annotation of Prokaryotic Taxa (FAPROTAX, version 1.2.7) database based on taxonomic assignments. Fungal ecological guilds were predicted using FUNGuild (version 1.1) by matching OTUs against reference taxa. Only predictions classified as “Probable” or “Highly Probable” confidence levels were retained. Since these approaches infer potential ecological functions based on taxonomic composition rather than direct measurements of microbial activity, the predicted functional profiles were interpreted as potential ecological functions. Relative abundances of functional groups were subsequently used for comparative analyses.

Differentially abundant microbial taxa were identified using linear discriminant analysis effect size (LEfSe), with a significance threshold of P < 0.05 and an LDA score > 3.0. Co-occurrence networks were constructed based on Spearman correlation coefficients (|r| > 0.6, P < 0.05), and network visualization and topological analyses were performed using Gephi (Zhu et al., 2025).

### 2.6 Statistical analysis

All statistical analyses were performed using SPSS 26.0, R software (version 4.3.1), Origin 2022, and SmartPLS 4.0. Data are presented as means ± standard deviations (SD). Before statistical analyses, data were tested for normality and homogeneity of variance. Statistical significance was defined as P < 0.05 unless otherwise specified.

Crop yield and nitrogen use efficiency (NUE) were analyzed using two-way analysis of variance (ANOVA), with year and fertilization treatment considered as fixed factors. Soil physicochemical properties and microbial alpha diversity indices were analyzed using one-way ANOVA followed by Fisher’s least significant difference (LSD) test for multiple comparisons.

Partial least squares structural equation modeling (PLS-SEM) was performed using SmartPLS 4.0 to investigate the relationships among soil properties, microbial communities, predicted microbial functional groups, and crop productivity. The model was constructed using 24 samples (n = 24). Latent variables were defined as follows: soil properties were represented by measured physicochemical parameters; microbial community composition was characterized using microbial diversity and community structure indices; predicted functional groups were derived from FAPROTAX and FUNGuild analyses; and crop productivity was represented by grain yield and nitrogen use efficiency (NUE).

Model reliability and validity were assessed using Cronbach’s alpha, composite reliability (CR), average variance extracted (AVE), and discriminant validity based on the Fornell-Larcker criterion. The explanatory capacity of endogenous variables was evaluated using the coefficient of determination (R^2^). Path coefficients were estimated using the partial least squares algorithm, and their significance was tested using a non-parametric bootstrap procedure with 5,000 resamples. Statistical significance was determined based on bootstrap-derived t-values and P-values (P < 0.05).

To account for multiple comparisons among microbial taxa and functional categories, P-values were adjusted using the Benjamini-Hochberg false discovery rate (FDR) correction.

## 3 Results and discussion

### 3.1 Results

#### 3.1.1 Effects of seasonal N carryover management on the performance of winter wheat–summer maize yield

Seasonal N carryover treatments significantly affected all measured yield components of winter wheat and summer maize (P < 0.05), with the exception of winter wheat TGW in 2022 (P > 0.05; **Supplementary Table 2**). For winter wheat, ear number and grain number per panicle were highest under W1M1 in 2022 (604 × 10^4^ ha^-1^ and 35.7, respectively), not significantly different from W1M0 (P > 0.05) but significantly higher than W0M1 and CK (P < 0.05); TGW did not differ significantly among treatments. Grain yield showed the same ranking, with W1M1 (10,040 kg ha^-1^) significantly exceeding all other treatments (P < 0.05). In 2023, ear number was highest under W1M0 (807 × 10^4^ ha^-1^) and W0M1 (790 × 10^4^ ha^-1^), both significantly exceeding W1M1 (732 × 10^4^ ha^-1^, P < 0.05), and TGW differed significantly among treatments, with W1M0 (44.7 g) the highest. Grain yield was highest under W1M0 (10,573 kg ha^-1^), not significantly different from W1M1 (9,841 kg ha^-1^, P > 0.05). For summer maize (**Table 2**), all yield components were highest under W1M1 in 2022, including ear number (6.33 × 10^4^ ha^-1^), grain number per panicle (476.0), TGW (382.3 g), and yield (11,520 kg ha^-1^), with no significant difference from W0M1 for most traits (P > 0.05). In 2023, ear number, grain number per panicle, and yield did not differ significantly between W0M1 and W1M1 (683 vs. 681 × 10^4^ ha^-1^; 561.6 vs. 556.1; 12,440 vs. 12,212 kg ha^-1^; P > 0.05), both significantly exceeding W1M0 and CK (P < 0.05); TGW did not differ significantly among treatments.

**Table 2:** Effects of different seasonal N carryover on annual yield of winter wheat-summer maize.

Year	Treatment	Winter wheat (kg ha^-1^)	Summer maize (kg ha^-1^)	Annual yield (t ha^-1^)
2022	CK	4750d	8150d	12.73d
	W1M0	9460b	9260c	18.72b
	W0M1	6550c	10900b	17.45c
	W1M1	10040a	11520a	21.56a
2023	CK	4855c	9929c	14.8b
	W1M0	10573a	10814b	21.4a
	W0M1	9269b	12440a	21.7a
	W1M1	9841ab	12212a	22.1a
Treatment		***	***	***
Year		**	NS	*
Year * Treatment		**	***	**

Different lowercase letters indicate significant differences in different treatments in the same year (*P* < 0.05). ‘ NS ‘ indicated no significant difference, ‘ * ‘ indicated significant difference (0.01 < *P* < 0.05), ‘ ** ‘ indicated extremely significant difference (0.001 < *P* < 0.01), ‘ * * * ‘ indicated very significant difference (*P* < 0.001). CK (control): no seasonal N carryover in both season; W1M0: N seasonal N carryover only during the winter wheat season; W0M1: N seasonal N carryover only during the summer maize season; and W1M1: N seasonal N carryover in both seasons.

Significant Year × Treatment interactions were detected for ear number, grain number per panicle, and yield in both crops (P < 0.05-0.001; **Supplementary Table 2**).

#### 3.1.2 Effects of different seasonal N carryover treatments on soil-crop system coupling coordination in winter wheat and summer maize season

The seasonal coupling coordination degree (D value) between wheat-soil and maize-soil systems showed clear temporal variations (**Figure 1**). During the wheat growing season, the coupling coordination degree remained at a low level (D < 0.2) across treatments, with a gradual decline observed in the 0–20 cm soil layer over time.

During the maize growing season, differences in coupling coordination degree among treatments were observed across growth stages. At the V6 stage, W1M0 showed relatively lower D values compared with W0M1 and W1M1. As the growing season progressed, these differences became less pronounced. From R3 to R6 stages, the coupling coordination degree under W1M0 showed a decreasing trend, resulting in comparatively lower values than W0M1 and W1M1 at maturity, particularly in the surface soil layer.

#### 3.1.3 Effects of different N efficiency indicators among the different N carryover seasons

Seasonal N carryover management substantially impacted annual NUE in the winter wheat-summer maize rotation system (**Figure 2**). When compared with W1M1, W1M0 and W0M1 increased the N partial factor productivity by 83.9% and 79.8% (**Figure 2B**), the N recovery efficiency by 87.5% and 0.8% (**Figure 2C**), and the agronomic efficiency by 61.4% and 51.4% (**Figure 2D**), respectively. Compared with W1M1, W1M0 and W0M1 exhibited higher APFPN and AEN, partially due to lower total annual N input.

**Figure 2:**
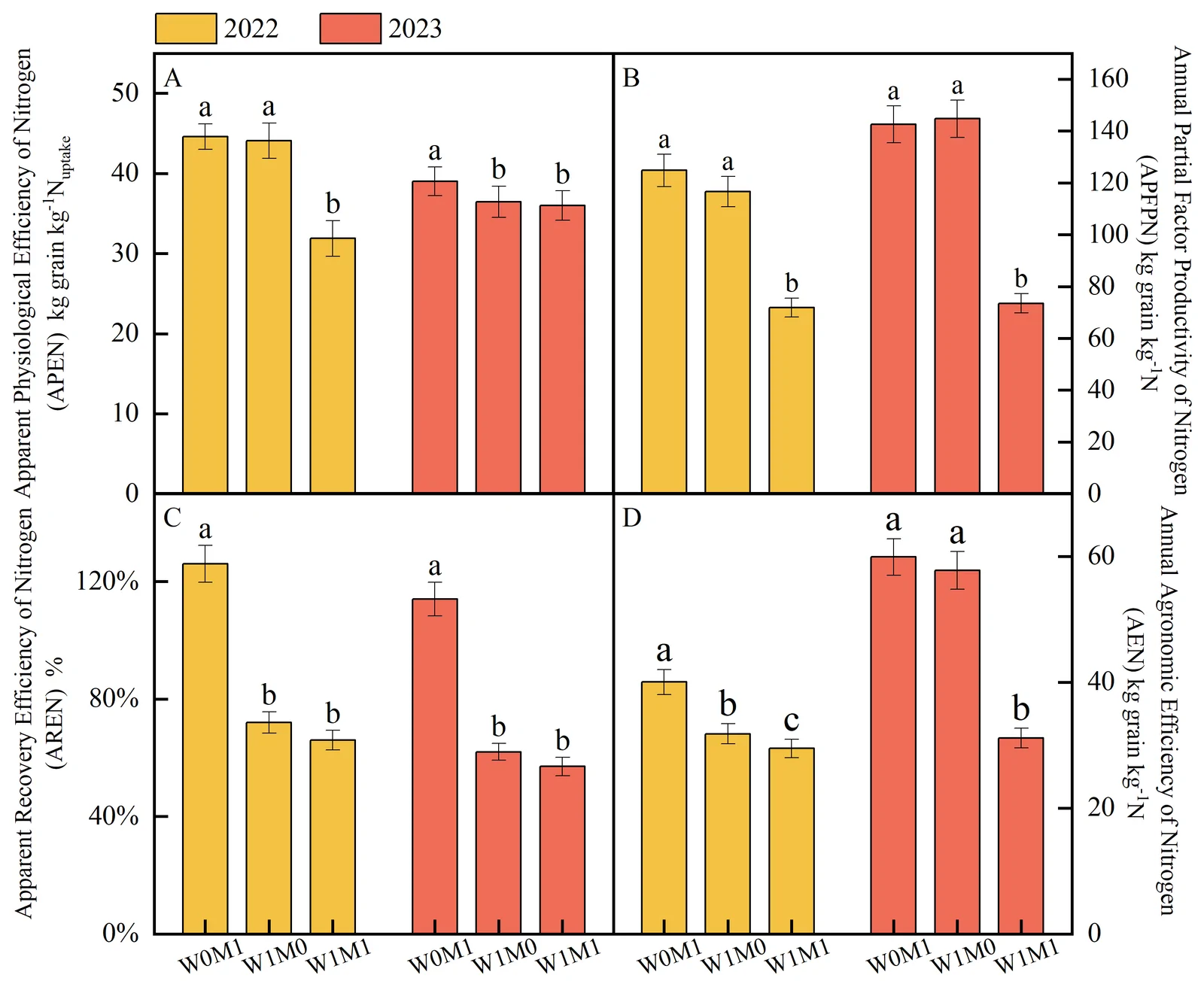
Effects of seasonal N carryover treatments on nitrogen use efficiency (NUE) in the winter wheat–summer maize rotation system. W1M0: N carryover only during the winter wheat season; W0M1: N carryover only during the summer maize season; W1M1: N carryover during both wheat and maize seasons. **(A)** Annual N physiological efficiency, **(B)** Annual N partial factor productivity, **(C)** Annual N recovery efficiency, **(D)** N agronomic efficiency.

#### 3.1.4 Effects of different seasonal N carryover treatments on soil microbial α- and β-diversity

##### 3.1.4.1 Effects of different seasonal N carryover treatments on soil microbial α-diversity

Alpha diversity analysis indicates that the effects of seasonal N carryover treatments on soil microbial communities vary across seasons and diversity indices (**Figure 3**). During the summer maize season, the Shannon index showed that bacterial diversity under W0M1 was significantly higher than that under W1M0. The Chao1 index showed no significant differences among treatments during the winter wheat season, while a significant difference was observed during the maize season between W0M1 and W1M0.

**Figure 3:**
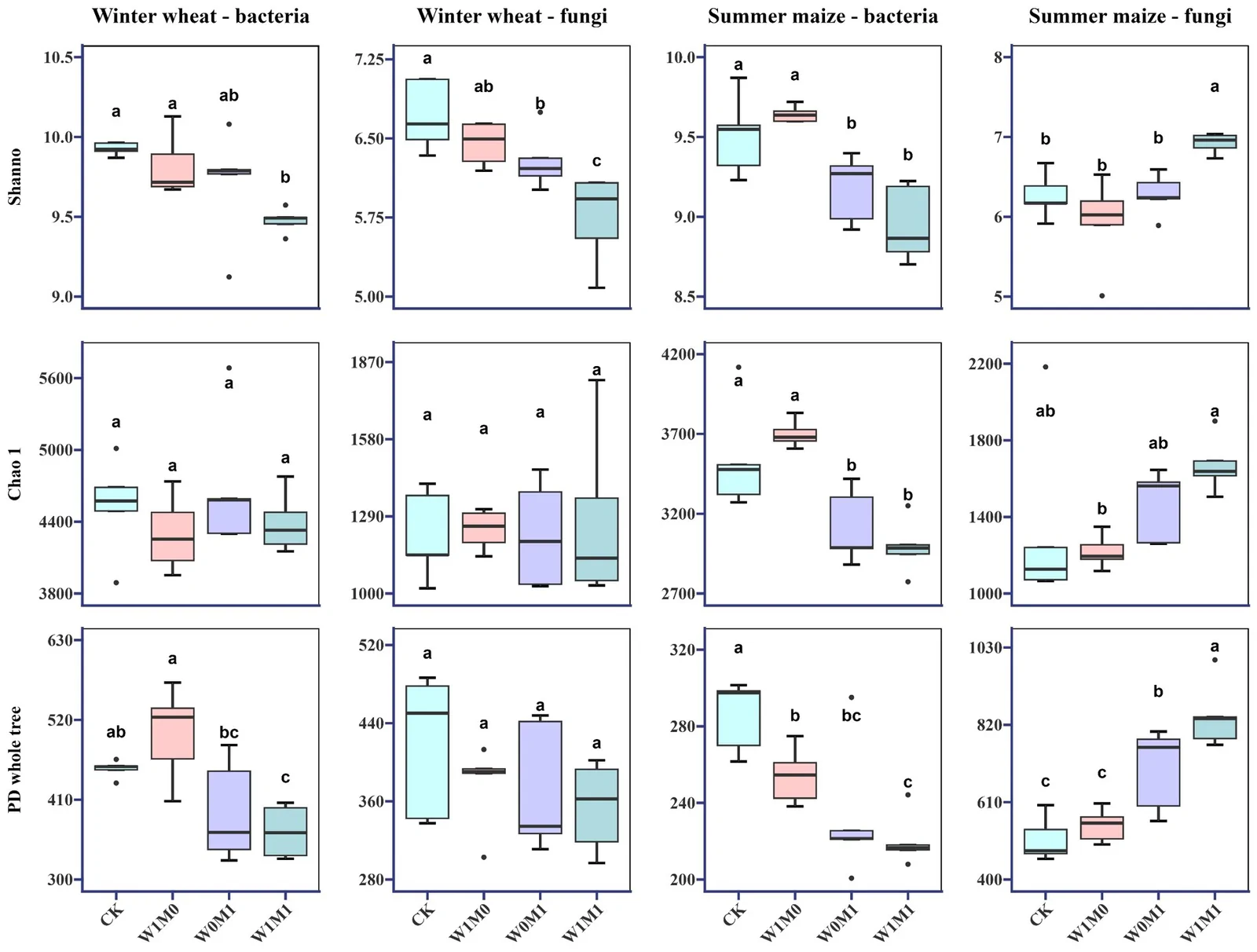
The α diversity index and significance test of bacteria and fungi in winter wheat-summer maize soil under different seasonal N carryover application. Values followed by a different small letter within a column are significantly different at 5% probability level. Shannon, Shannon index; Simpson, Simpson’s Diversity Index; PD whole tree, Faith’s phylogenetic diversity. CK, no N fertilizer applied during either wheat or maize seasons; W1M0, N carryover only during the winter wheat season; W0M1, N carryover only during the summer maize season; W1M1, N carryover during both wheat and maize seasons.

Faith’s phylogenetic diversity (PD whole-tree) further revealed contrasting seasonal patterns: bacterial phylogenetic diversity was higher under W1M0 compared with W0M1 and W1M1 during the wheat season, whereas W0M1 showed higher values than W1M0 during the maize season.

Overall, these results suggest that bacterial diversity responses were strongly dependent on both crop season and diversity metric. Fungal diversity indices also exhibited season-dependent and treatment-dependent variation, although response patterns differed among indices.

##### 3.1.4.2 Effects of different seasonal N carryover treatments on soil microbial β-diversity

Principal coordinate analysis (PCoA) based on the Bray-Curtis distance matrix (**Supplementary Figure 2**) showed that the first two axes explained 59.0% and 74.4% of the variation in fungal and bacterial communities during the winter wheat season, and 58.7% and 63.6% during the summer maize season, respectively, indicating that the ordination effectively captured the variation in microbial community composition.

ANOSIM analysis (**Supplementary Table 3**) further confirmed significant differences in bacterial community composition among fertilization treatments in both wheat and maize seasons (P < 0.05), with R values ranging from 0.696 to 1.000, indicating strong community separation. In contrast, fungal communities showed weaker separation (R = 0.176~0.864), with non-significant differences observed between several treatment comparisons (e.g., W1M1 and W0M1 and W1M1 and W1M0).

Overall, bacterial communities exhibited stronger treatment-induced compositional shifts than fungal communities, particularly during the maize season.

#### 3.1.5 Effects of different seasonal N carryover treatments on soil microbial community composition

##### 3.1.5.1 Effects of different seasonal N carryover treatments on soil microbial community composition at the phylum level

Through the analysis of soil microbial population abundance at the phylum level, it was found that different fertilization treatments significantly altered the composition of dominant microbial communities.

In the winter wheat season, the bacterial community was dominated by Pseudomonadota, Acidobacteriota, and unclassified bacteria, accounting for more than 50% of the total community (**Figure 4A**). Compared with the other fertilization treatments, W1M0 showed higher relative abundances of Bacillota (6.02%) and Actinobacteriota (7.60%). The relative abundance values reported here represent treatment abundances, and differences among treatments were evaluated statistically as shown in **Figure 4A**. Ascomycota was the dominant fungal phylum across all treatments, accounting for more than 60% of the fungal community (**Figure 4B**). W1M0 was associated with higher relative abundances of Blastocladiomycota (1.20%) and Basidiomycota (5.53%), whereas W0M1 showed a relatively higher abundance of Mucoromycota (2.27%).

**Figure 4:**
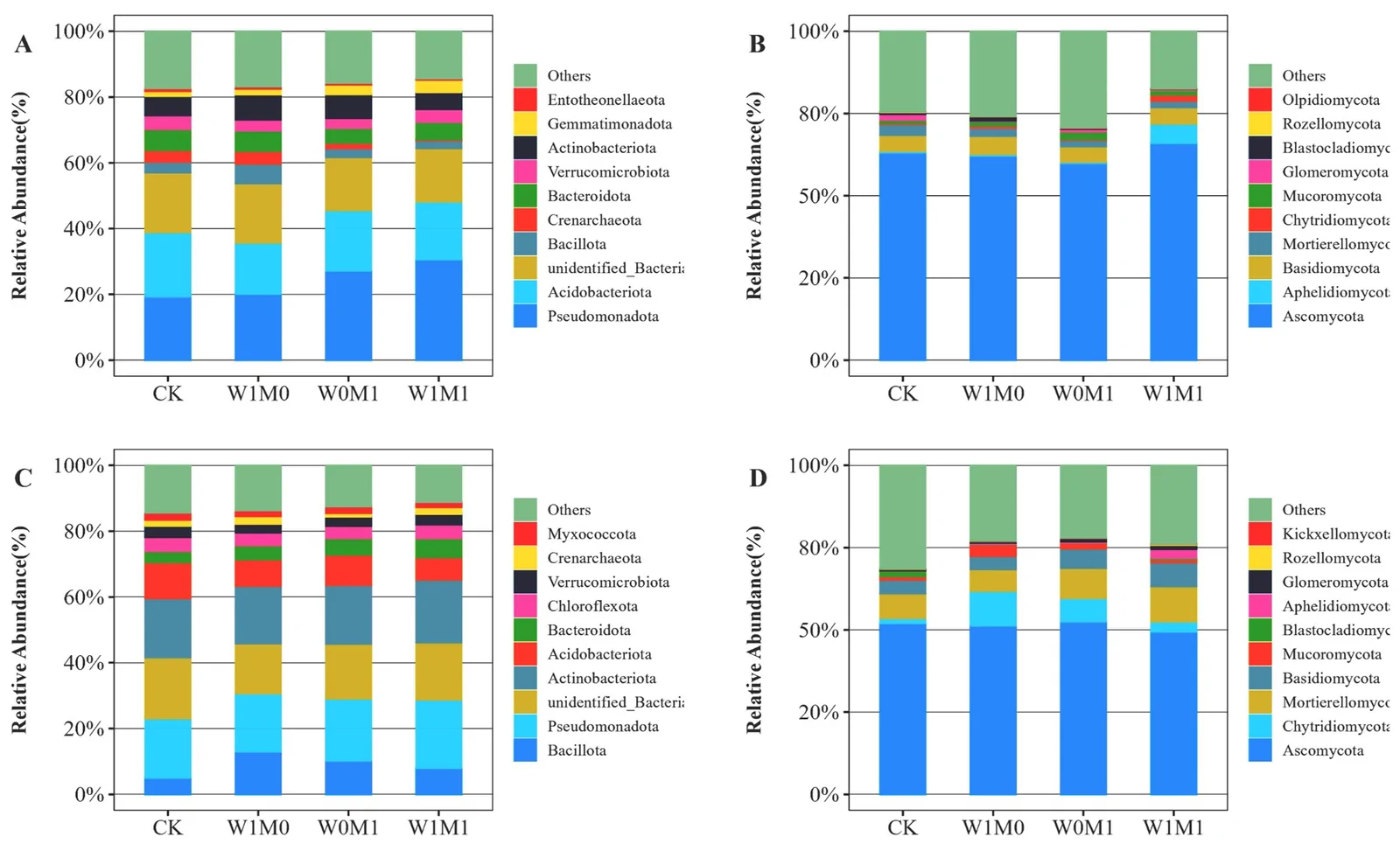
Different seasonal N carryover treatments on soil microbial community composition at phylum level. CK, no N fertilizer applied during either wheat or maize seasons; W1M0, N carryover only during the winter wheat season; W0M1, N carryover only during the summer maize season; W1M1, N carryover during both wheat and maize seasons. **(A, C)** represent the top 10 dominant bacterial species in wheat season and maize season, respectively. **(B, D)** represent the top 10 dominant fungal species in wheat season and maize season, respectively. Relative abundance < 1% is replaced by Others.

In the summer maize season, the bacterial community was mainly composed of Pseudomonadota, Acidobacteriota, Bacillota, Actinobacteriota, and Gemmatimonadota, which together accounted for more than 70% of the total bacterial community (**Figure 4C**). Compared with CK, all fertilized treatments increased the relative abundance of Bacillota (≥7.99% versus 5.04% in CK). Among the fertilized treatments, W1M0 exhibited relatively higher abundances of Bacillota (12.9%) and Crenarchaeota (2.38%). Ascomycota remained the dominant fungal phylum across treatments (**Figure 4D**). Differences among fertilization treatments were mainly reflected in the relative abundance of several minor fungal phyla, whereas the overall community composition remained broadly similar among treatments.

##### 3.1.5.2 Effects of different seasonal N carryover treatments on soil microbial community composition at the genus level

At the genus level, microbial community composition showed seasonal and treatment-dependent variation (**Supplementary Figure 4**).

During the winter wheat season, Sphingomonas and RB41 were among the dominant bacterial genera across treatments (**Supplementary Figure 4A**). Compared with CK, W1M0 showed higher relative abundances of several genera, including Allobaculum, Bifidobacterium, Dubosiella, and Lactobacillus, while RB41 decreased under W1M0. Fungal communities also showed treatment-dependent variation, with differences observed among Chaetomium, Cladorrhinum, Pseudogymnoascus, Sarocladium, and Lophotrichus across treatments (**Supplementary Figure 4B**).

During the summer maize season, Sphingomonas and RB41 remained dominant bacterial genera (**Supplementary Figure 4C**). W0M1 and W1M1 showed higher relative abundances of Sphingomonas and Lysobacter compared with CK, while RB41 decreased under fertilized treatments. Fungal communities were dominated by Cladosporium, Fusarium, Mortierella, and Botryotrichum (**Supplementary Figure 4D**), with treatment-specific differences observed among genera such as Cladosporium, Rhizophlyctis, and Mortierella.

Overall, genus-level community composition exhibited clear seasonal differences and treatment-dependent shifts.

#### 3.1.6 Effects of different seasonal N carryover treatments on the functional dynamics of soil microbial communities

Functional prediction analysis indicated that seasonal N carryover treatments altered the relative abundance of several predicted bacterial and fungal functional groups (**Figure 5**). Across both wheat and maize seasons, W1M0, W0M1, and W1M1 generally increased the relative abundance of taxa assigned to predicted chemoheterotrophy and aerobic chemoheterotrophy functions compared with CK (**Figures 5A, C**). Among the fertilized treatments, W1M1 showed the highest relative abundance of taxa associated with these predicted functions during the maize season.

**Figure 5:**
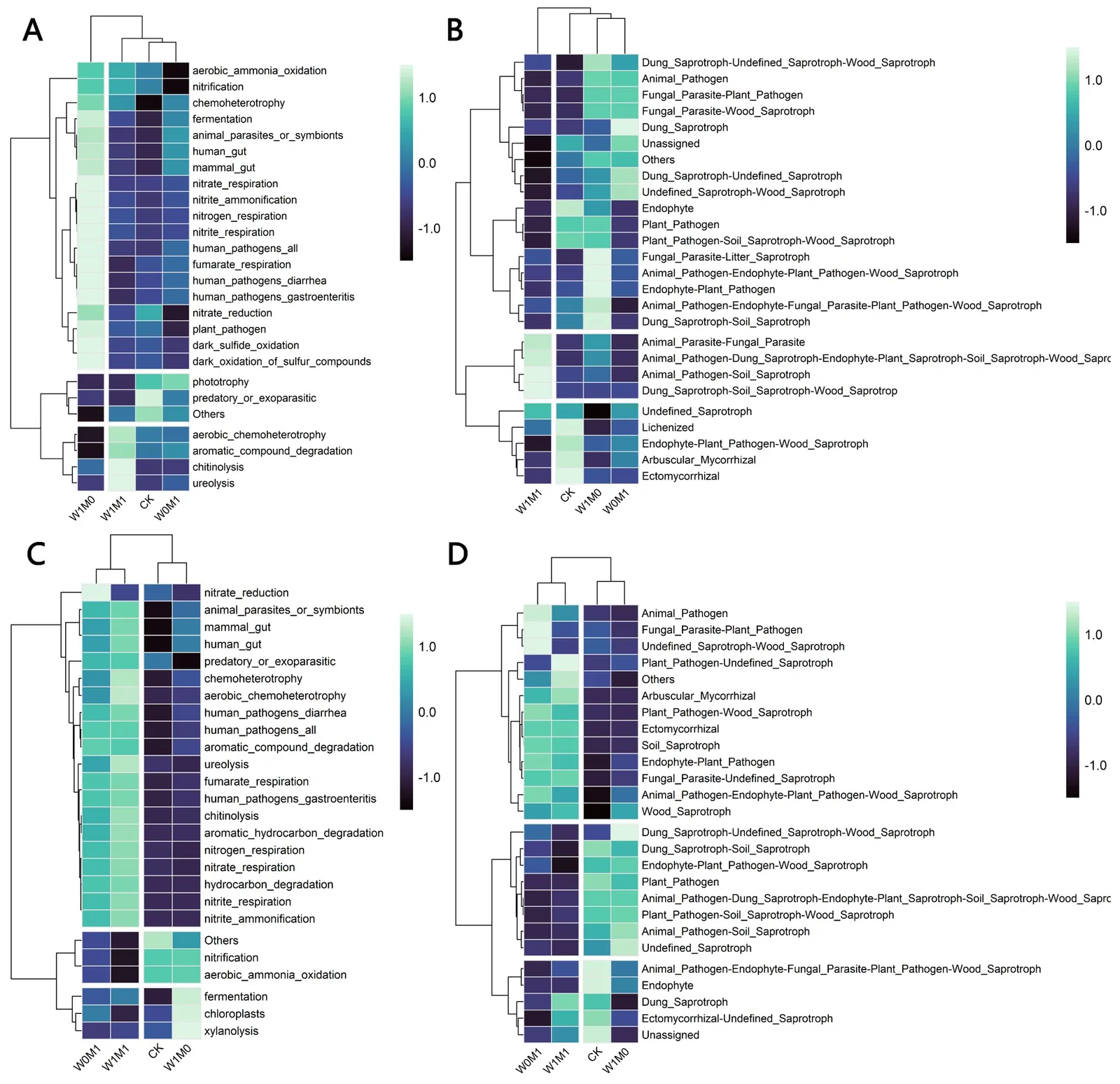
Functional cluster analysis of bacteria and fungi in winter wheat-summer maize soil under different seasonal N carryover application. CK, no N fertilizer applied during either wheat or maize seasons; W1M0, N carryover only during the winter wheat season; W0M1, N carryover only during the summer maize season; W1M1, N carryover during both wheat and maize seasons. **(A)** winter wheat season bacteria, **(B)** winter wheat season fungi, **(C)** summer maize season bacteria, **(D)** summer maize season fungi.

During the maize season, the relative abundance of taxa assigned to predicted nitrification-related functions was higher in CK and W1M0, whereas lower abundances were observed in W0M1 and W1M1 (**Figure 5C**). In contrast, taxa assigned to predicted denitrification and nitrate respiration-related functions were relatively more abundant in W0M1 and W1M1 than in CK and W1M0 (**Figure 5C**).

For fungal communities, taxa assigned to predicted arbuscular mycorrhizal and ectomycorrhizal guilds were relatively more abundant under W0M1 and W1M1 during the maize season (**Figure 5D**). In the wheat season, W1M0 showed the highest relative abundance of taxa assigned to fermentation-related functions (**Figure 5B**).

In addition, taxa assigned to FAPROTAX categories associated with human/animal gut and human-pathogen-related functions exhibited relatively higher abundances under W0M1 and W1M1 during the maize season (**Figure 5C**). These functional assignments were inferred from taxonomic information and do not represent direct measurements of microbial activity or pathogen occurrence.

#### 3.1.7 Effects of environmental factors on soil microbial communities under different seasonal N carryover treatments

##### 3.1.7.1 Correlation analysis between microorganisms and physical and chemical properties

Correlation analysis showed that in the maize season, Proteobacteria and Gemmatimonadota were enriched under W0M1 and W1M1 and positively correlated with available phosphorus and potassium (**Figure 6C**). Acidobacteriota was enriched in CK and negatively correlated with soil organic matter, total nitrogen, and yield. In the wheat season, Firmicutes and Bacteroidota, enriched under W1M0, positively correlated with pH and available phosphorus/potassium, respectively (**Figure 6A**). Acidobacteriota showed the strongest negative correlation with wheat yield, while Patescibacteria showed the strongest positive correlation. This seasonal reversal indicates that the key microbial phyla linked to productivity are season-dependent. At the genus level, Sphingomonas and Lysobacter positively correlated with available phosphorus and were consistently enriched under maize-season N carryover (**Figure 6D**). RB41 was enriched in CK and negatively correlated with yield. In the wheat season, Lactobacillus (enriched under W1M0) positively correlated with pH (**Figure 6B**). Nitrospira showed the strongest positive correlation with yield, highlighting nitrification as a key process specific to the wheat season, contrasting with the denitrification shift observed under maize-season carryover. Adhaeribacter and Marmoricola, which positively correlated with both nutrient availability and yield, are proposed as candidate soil-health indicators.

**Figure 6:**
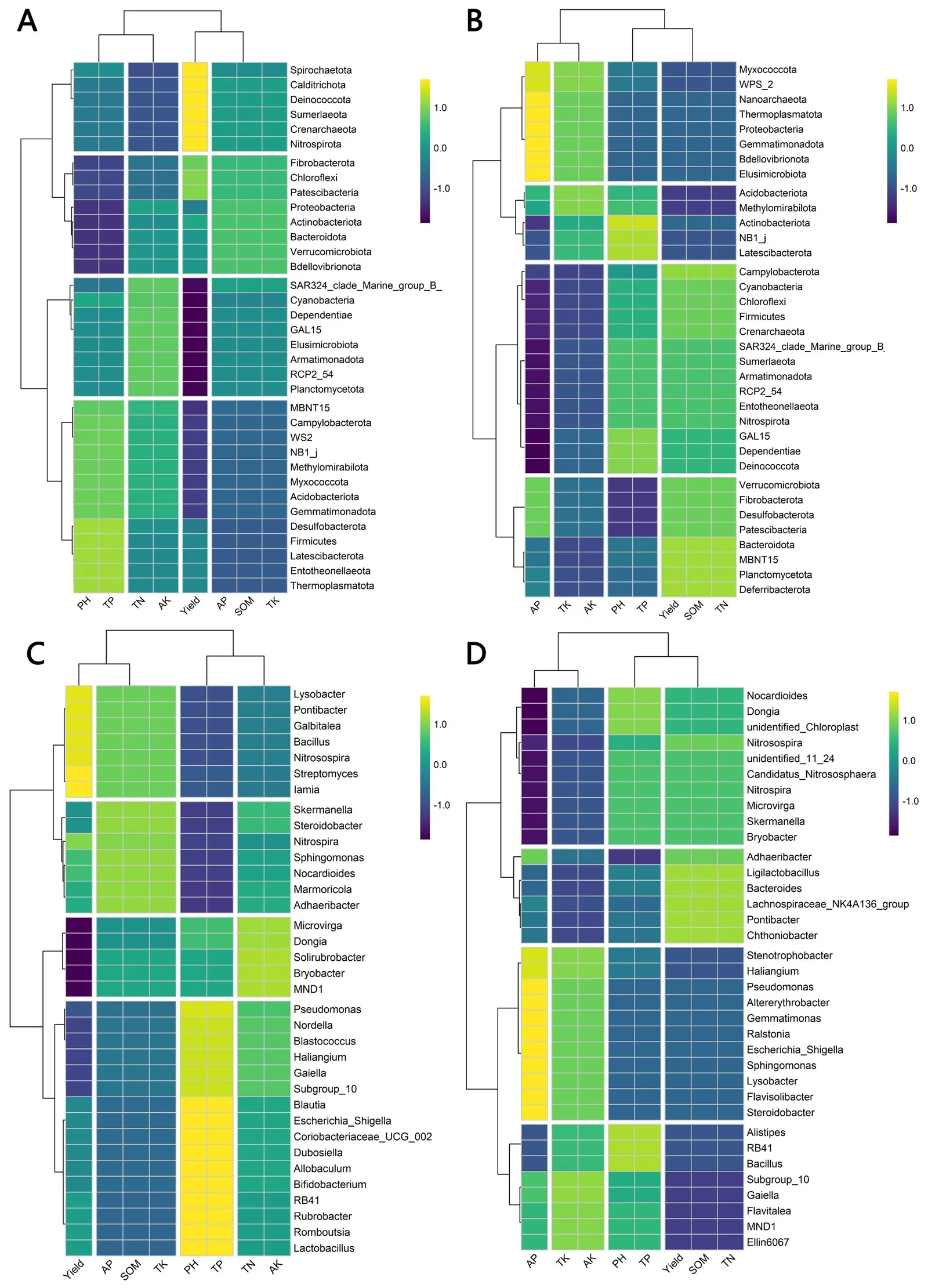
Effects of environmental factors on soil microbial community distribution at genus level and phylum level. **(A)** winter wheat season phylum level, **(B)** winter wheat season genus level, **(C)** summer maize season phylum level, **(D)** summer maize season genus level.

##### 3.1.7.2 Effects of environmental factors on the distribution of soil microbial communities

Redundancy analysis (RDA) revealed significant differentiation of bacterial and fungal communities during both wheat and maize seasons, with cumulative explanatory rates exceeding 80% (P < 0.01) (**Supplementary Figure 5**).

During the summer maize season, variation in bacterial community composition was primarily associated with pH, total phosphorus, and total potassium, whereas soil organic matter and available potassium were negatively associated with community distribution. Fungal community variation was mainly associated with pH and available phosphorus. During the winter wheat season, bacterial community composition was associated with pH, total nitrogen, and total potassium, whereas fungal community composition was associated with available phosphorus, total nitrogen, and total potassium.

The ordination patterns indicated that microbial community variation was significantly associated with soil pH and nutrient-related variables. During the summer maize season, W0M1 and W1M1 were associated with higher pH and phosphorus-potassium nutrient status, whereas differences among fertilization treatments during the wheat season were associated with variation in soil nitrogen, phosphorus, and potassium availability. Across seasons, pH and phosphorus- and potassium-related variables were consistently associated with microbial community composition, whereas total nitrogen showed stronger associations with fungal community distribution.

### 3.2 Discussion

#### 3.2.1 Crop yield and nitrogen use efficiency in response to seasonal nitrogen legacy

The differences in yield and nitrogen use efficiency among treatments are consistent with previous findings in wheat-maize rotation systems. Imbalanced seasonal nitrogen supply has been reported to be associated with reduced system-level nitrogen use efficiency, whereas coordinated nitrogen management across seasons is associated with improved crop performance (Wang et al., 2019; Ma et al., 2020). In addition, limited recovery of seasonal N carryover in subsequent crops has been widely reported, indicating that only a fraction of residual soil nitrogen can be utilized within a single rotation cycle (Wang et al., 2019).

The relatively higher contribution of W0M1 to annual yield is consistent with previous studies showing strong synchronization between maize nitrogen demand and fertilizer nitrogen supply during key growth stages such as tasseling and grain filling (Yan et al., 2019). Moreover, excessive or mismatched nitrogen supply has been reported to be associated with reduced nutrient translocation and lower yield formation efficiency during reproductive stages (Feng et al., 2024; Hammad et al., 2022). Overall, these findings suggest that temporal matching between nitrogen supply and crop demand is more important than total nitrogen input in regulating yield and nitrogen use efficiency in rotation systems (Chen et al., 2024).

#### 3.2.2 Seasonal nitrogen management alters soil-crop system coupling coordination

The responses of soil microbial communities to seasonal nitrogen carryover treatments are consistent with previous studies in agroecosystems. Seasonal nitrogen inputs have been shown to be associated with changes in microbial community composition and diversity (Li et al., 2021; Niu et al., 2021). In addition, bacterial communities generally appear more sensitive to fertilization than fungal communities, likely due to differences in ecological strategies and environmental adaptability (Zandt et al., 2023; Murindangabo et al., 2023).

#### 3.2.3 Divergent responses of bacterial and fungal communities to seasonal N carryover

The observed enrichment of Bacillota and Actinobacteriota under fertilized treatments is consistent with their reported roles in organic matter decomposition and nutrient cycling in agricultural soils (Tagele et al., 2023). Previous studies have also suggested that fertilization may increase microbial functional redundancy while simultaneously altering community composition (Tian et al., 2022).

Reductions in microbial diversity under certain treatments should not be directly interpreted as ecosystem instability, as diversity shifts may also reflect resource-driven community restructuring rather than functional degradation (Li et al., 2018; Niu et al., 2021). LEfSe analysis identified several treatment-associated taxa (**Supplementary Figure 3**), indicating that seasonal nitrogen management was associated with distinct shifts in microbial community composition between treatments and seasons.

#### 3.2.4 Predicted functional shifts and their ecological implications

The predicted functional shifts observed in this study are broadly consistent with previous research on nitrogen-driven changes in microbial functional potential (Anand and Rajeshkumar, 2022). Increased relative abundance of chemoheterotrophic functions under fertilization has been widely reported and is generally associated with enhanced substrate availability in agricultural soils (Zhou et al., 2016; Shi et al., 2023).

Changes in predicted nitrification and denitrification related functional groups are consistent with previous findings that nitrogen inputs are associated with shifts in soil nitrogen transformation potential (Xing et al., 2025). In addition, responses of mycorrhizal fungi to nutrient availability have been widely reported, with phosphorus-nitrogen interactions playing an important role in regulating symbiotic fungal associations (Xu et al., 2018).

Furthermore, seasonal nitrogen carryover has been reported to be associated with changes in microbial nitrogen transformation potential and carbon-nitrogen coupling processes, which is consistent with the functional differences observed among treatments in this study (Chang et al., 2024).

#### 3.2.5 PLS-SEM reveals treatment-specific soil-microbe-crop association patterns

Overall, the integrated PLS-SEM modeling revealed that soil physicochemical properties serve as the strongest and most consistent direct driver of crop productivity, regardless of seasonal nitrogen allocation. Across all treatment sub-models, the standardized path coefficient from soil physicochemical properties to crop yield consistently exceeded 0.900 (e.g., β = 0.995 in W0M1-wheat, β = 0.939 in W1M0-maize, β = 0.922 in CK-maize), with crop yield variance explained (R^2^) ranging from 0.539 to 0.762 (**Figure 7**). Soil nitrogen content acts as the primary regulator of soil physicochemical properties, explaining a substantial proportion of their variance (R^2^ ranging from 0.368 to 0.975), particularly under single-season fertilization or unfertilized regimes (**Figure 7**). Regarding microbial communities, bacterial communities exhibited stronger structural associations with soil physicochemical properties (e.g., β = 0.981 in CK-maize) than fungal communities, although their direct contributions to yield were consistently lower than that of the soil physicochemical properties (**Figure 7E**). Fungal communities displayed weaker and more variable correlations with both soil variables and crop yield across all models, indicating their limited role as direct predictors in this system (Xiang et al., 2020).

**Figure 7:**
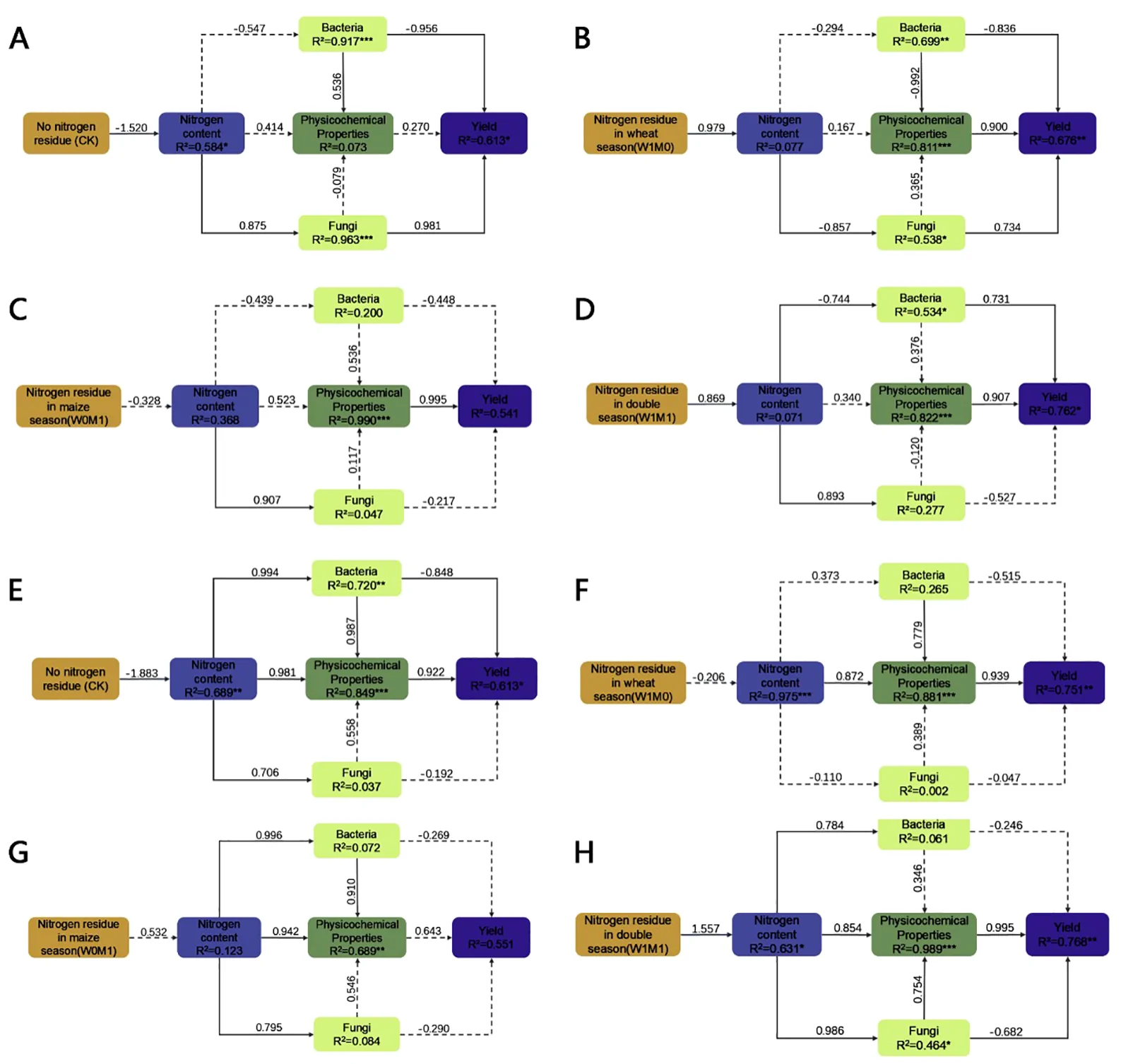
Structural equation model of the effect of seasonal N carryover on soil properties and microbial yield in summer maize and winter wheat season. CK, no N fertilizer applied during either wheat or maize seasons; W1M0, N carryover only during the winter wheat season; W0M1, N carryover only during the summer maize season; W1M1, N carryover during both wheat and maize seasons. **(A–D)** depict the models for each treatment during the wheat season, while **(E–H)** show the models for each treatment during the maize season.

A distinct difference in the magnitude and mechanistic pathway of legacy effects was observed between W1M0 (nitrogen applied in the wheat season, influencing the subsequent maize season) and W0M1 (nitrogen applied in the maize season, influencing the subsequent wheat season). Specifically, the W1M0 treatment demonstrated a significantly stronger and more direct carryover effect, as soil nitrogen content from the preceding wheat season explained 97.5% of the variance in soil physicochemical properties of the subsequent maize season (R^2^ = 0.975) with a strong direct path coefficient of 0.872, which in turn drove crop yield with a path coefficient of 0.939 (**Figure 7F**). In contrast, the residual effect of W0M1 on the following wheat season was notably weaker and more complicated; its soil nitrogen explained only 36.8% of the variance in the subsequent wheat soil properties (R^2^ = 0.368) with a lower path coefficient of 0.523, and even exhibited a negative path coefficient of -0.328 directly linking nitrogen to yield (**Figure 7C**). Quantitatively, the explanatory power of soil nitrogen for the subsequent soil properties under W1M0 was approximately 2.65 times greater than that under W0M1, indicating that nitrogen applied during the wheat season produces a profoundly more efficient and positive legacy on soil-crop coordination than nitrogen applied in the maize season.

Overall, the PLS-SEM results suggest that the strength and direction of associations among soil properties, microbial communities, and yield varied across fertilization treatments. These results should be interpreted as multivariate statistical relationships rather than causal mechanisms (Ahmad et al., 2023). Similar soil-microbe-crop interaction relationships have been reported in previous studies, where soil nutrient availability was identified as a key driver of microbial community structure and crop productivity (Zhou et al., 2016; Shi et al., 2023). However, these relationships should be interpreted as statistical associations rather than direct causal mechanisms. These functional predictions should be interpreted as inferred potential shifts based on taxonomic composition, rather than direct evidence of microbial metabolic processes.

However, all functional results are based on taxonomic inference rather than direct measurements of microbial activity. Therefore, these results represent predicted functional potential rather than confirmed functional changes, consistent with limitations of predictive functional profiling approaches (Niu et al., 2021).

## 4 Conclusions

Seasonal shifts in microbial community composition were closely tied to residue management and crop productivity. In the maize season, W0M1 (maize residue only) and W1M1 (both wheat and maize residues) similarly enriched Proteobacteria and Gemmatimonadota, whereas Acidobacteriota dominated in the unamended control. The wheat season presented a contrasting pattern: W1M0 (wheat residue only) specifically enriched Firmicutes and Bacteroidota, while Acidobacteriota again showed negative associations with yield, and Patescibacteria emerged as the strongest positive correlate. Correlation analyses further revealed that Sphingomonas and Lysobacter were consistently linked to available phosphorus under maize-season nitrogen carryover, whereas RB41 (enriched in CK) was negatively associated with yield. In the wheat season, Lactobacillus (enriched under W1M0) correlated with pH, and Nitrospira showed the strongest positive yield correlation, pointing to nitrification as a wheat-specific process-in contrast to the denitrification shift observed under maize-season carryover. Collectively, these seasonal reversals highlight that key microbial taxa associated with productivity depend on crop season and residue type. Notably, Adhaeribacter and Marmoricola, which correlated positively with both nutrient availability and yield across seasons, are proposed as candidate soil-health indicators.

## Data Availability

The datasets presented in this study can be found in online repositories. The names of the repository/repositories and accession number(s) can be found below: NCBI Sequence Read Archive (SRA), BioProject accession numbers PRJNA1522895 (bacterial 16S rRNA amplicon sequencing) and PRJNA1522898 (fungal ITS amplicon sequencing), available at https://www.ncbi.nlm.nih.gov/bioproject/PRJNA1522895 and https://www.ncbi.nlm.nih.gov/bioproject/PRJNA1522898, respectively.
