# Supplementary material for: Season-specific nitrogen management shapes soil microbial communities and crop productivity in wheat–maize rotation system
Source: Front Plant Sci. 2026 Sep 16;17:1938392. doi: 10.3389/fpls.2026.1938392 (PMC13624160; doi:10.3389/fpls.2026.1938392)
Supplement: Supplementary file 1 [file DataSheet1.docx]

**1. Figure**

**Fiig S1 Monthly Average Precipitation and Temperature for 2022–2023**

This is used in Section 2.1.

Fig S2 PCoA of bacteria and fungi in winter wheat-summer maize soil under seasonal N carryover treatments. CK: no N fertiliser applied during either wheat or maize seasons; W1M0: N carryover only during the winter wheat season; W0M1: N carryover only during the summer maize season; W1M1: N carryover during both wheat and maize seasons. Phosphate and potash fertilisers were applied in all treatments. Fig A: winter wheat season bacteria, Fig B: winter wheat season fungi, Fig C: summer maize season bacteria, Fig D: summer maize season fungi.

This figure is used in the section 3.1.4, results of PCoA of bacteria and fungi in winter wheat-summer maize soil under different seasonal N carryover treatments.

**Fig S3 LEfSe analysis of microbial community differences in phylum levels among different seasonal N carryover treatments**. CK: no N fertiliser applied during either wheat or maize seasons; W1M0: N carryover only during the winter wheat season; W0M1: N carryover only during the summer maize season; W1M1: N carryover during both wheat and maize seasons. Phosphate and potash fertilisers were applied in all treatments. Fig A: differential soil bacterial in winter wheat season, Fig B: differential soil fungal in winter wheat season, Fig C: differential soil bacterial in summer maize season, Fig D: differential soil fungal in summer maize season.

This figure is used in the section 3.1.5 part 2, results of differences in microbial communities between LEfSe analysis groups treated in different seasonal N carryover.

 Fig S4 Effects of seasonal N carryover treatments on soil microbial community composition at genus level. CK: no N fertiliser applied during either wheat or maize seasons; W1M0: N carryover only during the winter wheat season; W0M1: N carryover only during the summer maize season; W1M1: N carryover during both wheat and maize seasons. Phosphate and potash fertilisers were applied in all treatments. Fig A: soil bacterial community in winter wheat season, Fig B: soil fungal community in winter wheat season, Fig C: soil bacterial community in summer maize season, Fig D: soil fungal community in summer maize season.

This figure is used in the section 3.1.5 part 2, results of Effects of different seasonal N carryover on soil microbial community composition at genus level.

Fig S5 RDA analysis of seasonal N carryover treatments application seasons. CK: no N fertiliser applied during either wheat or maize seasons; W1M0: N carryover only during the winter wheat season; W0M1: N carryover only during the summer maize season; W1M1: N carryover during both wheat and maize seasons. Phosphate and potash fertilisers were applied in all treatments. Fig A: soil bacterial community in winter wheat season, Fig B: soil fungal community in winter wheat season, Fig C: soil bacterial community in summer maize season, Fig D: soil fungal community in summer maize season.

This figure is used in the section 3.7.2.

**2.table**

This table is intended to illustrate the fundamental geographical context prior to the commencement of the experiment (section 2.1).

**Table S1 Physicochemical properties of soil in experimental site**

| **Treatment** | **pH** | **SOM** | **Total N** | **Total P** | **Total K** | **AP** | **AK** |
| --- | --- | --- | --- | --- | --- | --- | --- |
|  |  | (g kg^-1^) | (g kg^-1^) | (g kg^-1^) | (g kg^-1^) | (mg kg^-1^) | (mg kg^-1^) |
| **CK** | 7.735 | 9.037 | 1.065 | 1.057 | 8.032 | 7.882 | 8.042 |
| **W1M0** | 7.642 | 9.047 | 0.985 | 0.977 | 7.912 | 8.002 | 8.022 |
| **W0M1** | 7.721 | 8.912 | 1.035 | 1.017 | 7.992 | 8.012 | 7.982 |
| **W1M1** | 7.752 | 8.981 | 0.995 | 1.047 | 7.952 | 7.952 | 7.952 |

CK: no N fertiliser applied during either wheat or maize seasons; W1M0: N carryover only during the winter wheat season; W0M1: N carryover only during the summer maize season; W1M1: N carryover during both wheat and maize seasons. Phosphate and potash fertilisers were applied in all treatments..

This table is used in the section 3.1 Used to indicate the yield of wheat-maize rotation after two years.

**Table S2 Effects of different seasonal N carryover on yield components of winter wheat-summer maize**

| **Season** | | **Winter wheat** | | | | **Summer maize** | | | |
| --- | --- | --- | --- | --- | --- | --- | --- | --- | --- |
| **Year** | **Treatment** | **Hectares ear number** | **Grain number per panicle** | **Thousand-grain weight** | **Yield**  (kg ha^-1^) | **Hectares ear number** | **Grain number per panicle** | **Thousand-grain**  **weight**  (g) | **Yield**  (kg ha^-1^) |
|  |  | (10^4^ ha^-1^) |  | (g) |  | (10^4^ ha^-1^) |  |  |  |
| **2022** | **CK** | 499c | 20.1c | 45.7a | 4570d | 5.5c | 416.0c | 354.5c | 8150d |
|  | **W1M0** | 583ab | 33.2a | 49.1a | 9460b | 5.8b | 424.6bc | 371.9b | 9260c |
|  | **W0M1** | 523bc | 26.4b | 47.4a | 6550c | 6.2a | 455.6ab | 380.7a | 10900b |
|  | **W1M1** | 604a | 35.7a | 46.5a | 10040a | 6.33 a | 476.0a | 382.3a | 11520a |
| **2023** | **CK** | 573c | 21.3c | 39.7c | 4855c | 619b | 500.1b | 314.5a | 9929c |
|  | **W1M0** | 807a | 29.3b | 44.7a | 10573a | 631b | 516.2b | 319.6a | 10814b |
|  | **W0M1** | 790a | 29.0b | 40.5c | 9269b | 683a | 561.6a | 324.2a | 12440a |
|  | **W1M1** | 732b | 32.2a | 41.8b | 9841ab | 681a | 556.1a | 322.6a | 12212a |
| **Treatment** | | *** | *** | NS | *** | *** | *** | * | *** |
| **Year** | | ** | * | *** | ** | ** | ** | ** | NS |
| **Year * Treatment** | | ** | ** | * | ** | * | *** | NS | *** |

Different lowercase letters indicate significant differences in different treatments in the same year (*P* < 0.05). ' NS ' indicated no significant difference, ' * ' indicated significant difference (0.01 < P <0.05), ' * * ' indicated extremely significant difference (0.001 < P<0.01), ' * * * ' indicated very significant difference (*P* < 0.001). CK: no N fertiliser applied during either wheat or maize seasons; W1M0: N carryover only during the winter wheat season; W0M1: N carryover only during the summer maize season; W1M1: N carryover during both wheat and maize seasons. Phosphate and potash fertilisers were applied in all treatments.

This table is used in the section 3.4.2 Used to indicate the difference of wheat-maize rotation

**Table S3 ANOSIM of bacteria and fungi in winter wheat-summer maize soil under different seasonal N carryover treatments.**

| Contrast | Wheat-Bacteria | | Wheat-Fungi | | Maize-Bacteria | | Maize-Fungi | |
| --- | --- | --- | --- | --- | --- | --- | --- | --- |
|  | R | P | R | P | R | P | R | P |
| W0M1-CK | 0.420 | 0.011 * | 0.820 | 0.011 * | 0.604 | 0.010 * | 1.000 | 0.004 ** |
| W1M0-CK | 0.560 | 0.012 * | 0.808 | 0.005 * | 0.396 | 0.011 * | 0.212 | 0.031 * |
| W1M1-CK | 1.000 | 0.005 ** | 0.948 | 0.010 * | 0.696 | 0.011 * | 0.704 | 0.012 * |
| W0M1-W1M0 | 0.308 | 0.042 * | 0.384 | 0.035 * | 1.000 | 0.009 ** | 0.864 | 0.007 ** |
| W1M1-W0M1 | 0.600 | 0.007 ** | 0.468 | 0.014 * | 0.732 | 0.006 ** | 0.176 | 0.050 NS |
| W1M1-W1M0 | 0.820 | 0.006 ** | 0.624 | 0.009 ** | 1.000 | 0.009 ** | 0.328 | 0.077 NS |

CK: no N fertiliser applied during either wheat or maize seasons; W1M0: N carryover only during the winter wheat season; W0M1: N carryover only during the summer maize season; W1M1: N carryover during both wheat and maize seasons. Phosphate and potash fertilisers were applied in all treatments. ' NS ' indicated no significant difference, ' * ' indicated significant difference (0.01 < *P* <0.05), ' * * ' indicated extremely significant difference (0.001 < *P* <0.01), ' * * * ' indicated very significant difference (*P* < 0.001).
